# Association between gastro-intestinal symptoms and menstruation in patients with ileal pouches

**DOI:** 10.1093/gastro/gou042

**Published:** 2014-07-12

**Authors:** Shishira Bharadwaj, Xian-rui Wu, Matthew D. Barber, Elaine Queener, Lesley Graff, Bo Shen

**Affiliations:** ^1^Departments of Gastroenterology/Hepatology, Cleveland Clinic Foundation, Cleveland, Ohio, USA; ^2^Colorectal surgery, Cleveland Clinic Foundation, Cleveland, Ohio, USA and ^3^Obstetrics and Gynecology, the Cleveland Clinic Foundation, Cleveland, Ohio, USA and ^4^Department of Clinical Health Psychology, University of Manitoba, Winnipeg, Manitoba, Canada

**Keywords:** inflammatory bowel disease, ileal pouch-anal anastomosis, menstruation, symptomatology, restorative proctocolectomy

## Abstract

**Background and aims:** Gastro-intestinal (GI) symptoms are often experienced by healthy women during menstruation. An increased frequency of GI symptoms during menses has also been reported in women with irritable bowel syndrome or inflammatory bowel disease (IBD); however, IBD patients with restorative proctocolectomy and ileal pouch-anal anastomoses (IPAA) have not been studied. We aimed to examine the association between GI symptoms before and during menses in patients with IPAA, and to assess factors for exacerbation of GI symptoms in those patients.

**Methods:** Adult women recorded in the Pouchitis Registry were invited to participate in a mailed survey. Participants reported on GI symptoms 1–5 days prior to- (pre-menses) and during the days of their menses in recent months. Demographic and clinical variables were obtained through the survey and chart review.

**Results:** One hundred and twenty-eight (21.3%) out of 600 women with IPAA responded to the survey questionnaire. Forty-three (33.5%) were excluded for reasons including post-menopausal (*n = *25), hysterectomy (*n = *14) and use of contraceptives (*n = *4). Abdominal pain (*P = *0.001), diarrhea (*P = *0.021), and urgency (*P = *0.031) were more commonly reported during menses than pre-menses by the participants. Only a history of painful menses was significantly associated with increased GI symptoms during menses for patients with ileal pouch (odds ratio = 5.67; 95% confidence interval: 1.41–22.88; *P = *0.015).

**Conclusion:** GI symptoms such as abdominal pain, diarrhea, and urgency are commonly associated with menses in patients with ileo-anal pouch. Painful menses may be associated with worsening of GI symptoms.

## INTRODUCTION

Several events during women’s lives cause fluctuations in hormone levels, including puberty, use of oral contraceptive pills (OCP’s), pregnancy, lactation and menopause [1]. Fluctuations in hormonal levels also affect non-gynecological systems, such as the gastro-intestinal (GI) tract, due to the presence of steroid receptors in the GI tract [2]. Results from both prospective and retrospective studies focusing on GI transit in healthy individuals during the menstrual cycle have been inconclusive, although a few studies have shown increased transit time during the luteal phase [1, 3]. Further support of hormonal response on GI tract comes from the effect of progesterone during pregnancy, which includes gastro-esophageal reflux, constipation and gall bladder dysfunction [2].

Although GI symptom changes during the menstrual cycle are observed in healthy women, patients with irritable bowel syndrome (IBS) more commonly reported symptoms, particularly during menstruation [6]. This may result from increased perception of viscera-somatic stimuli and increased abdominal distension, pain and bloating due to an elevated level of menstrual prostaglandin (PG) levels [3]. Further, evidence of the role of sex hormones in the generation of GI symptoms is derived from studies showing therapeutic relief of IBS symptoms following hysterectomy and use of gonadotropin-releasing hormone analog [4].

Recent studies have extrapolated the above findings and further investigated the variation in GI symptoms in patients with inflammatory bowel disease (IBD) [5, 6]. There is general perception that women with IBD experience heightened symptoms during menses, with diarrhea being more common than constipation [10]. However, results from those studies have been inconclusive on whether the worsen symptomatology is normal physiological exacerbation versus disease exacerbation. Restorative proctocolectomy with ileal pouch anastomosis (IPAA) has been the standard of care in ulcerative colitis (UC) patients following colectomy [7]. Restorative proctocolectomy with IPAA has been shown to improve patients' health-related quality of life and lead to possible discontinuation of UC-related medications and reduction in the risk of UC–associated neoplasia [12]. Variation in GI symptoms during menstrual cycle in IBD patients with IPAA has not been studied. We hypothesized that patients with IPAA may have exacerbation of GI symptoms during menses.

We aimed to study the association between GI symptoms, before and during menses, in patients with IPAA and to assess factors for GI symptom exacerbation in those patients.

## MATERIALS AND METHODS

### Participants and process

From 2000 to 2012, 1221 patients were seen at our sub-specialty pouchitis clinic. Adult women were identified from the Pouchitis Registry database maintained by the clinic, and were invited to participate in the study if they met the following inclusion criteria: (i) age between 18 and 65 years. The reason to extend the upper age limit to 65 was to ensure a higher response rate, (ii) underlying IBD and (iii) IPAA. Exclusion criteria were (i) patients with IPAA for familial adenomatous polyposis or colon neoplasm or (ii) secondary dysmenorrhea, known endometriosis, and adenomyosis. Eligible participants (*n = *600) were mailed a survey and a pre-addressed, stamped envelope to return the information. Electronic medical records were reviewed to extract additional demographic information and clinical variables. The Cleveland Clinic Institutional Review Board approved the study, and informed consent was obtained from all participants.

One hundred and twenty-eight (21.3%) women responded to the mailed questionnaire. Forty three (33.5%) were excluded for reasons including menopausal who partially completed the questionnaire (*n = *25), hysterectomy (*n = *14), and use of contraceptives (*n = *4). The patients were further divided into two groups based on symptom score of the validated modified Pouchitis Disease Activity Index (mPDAI) score [8, 9]: the study group (GI symptom exacerbation during the menses, *n = *46) and control group (stable or GI symptoms better during menses, *n = *39) to assess the factors for worsening GI symptoms during menses. A subgroup of post-menopausal women who completed the survey (*n = *8; 9.4%) was also included in the main analysis.

### Demographic and clinical variables

Background, disease-related and gynecological variables were evaluated in the study. Background demographic and health history variables included age, education level, ethnic background, body mass index (BMI), history of smoking, excessive alcohol use, health insurance, and use of non-steroidal anti-inflammatory drugs (NSAIDs). Pouch-related disease variables included family history of IBD and colon cancer, diagnosis of underlying disease (UC or indeterminate colitis), duration of IBD, extent of IBD, indication for colectomy, presence of fulminant colitis, configuration of ileal pouch, stage of pouch surgery, duration of pouch, pouch diagnosis, current use of pouch medications, and extra-intestinal manifestations. Gynecological variables included age at first menses, length of menstrual cycle, days of menstrual flow, whether menses are usually painful, inter-menstrual bleeding, number of pregnancies, number of vaginal deliveries, number of caesarean sections, number of children, use of OCP’s, and menopausal status. Concurrent medication use was also documented from questionnaires, including aspirin, 5-aminosalicylates, antibiotics, corticosteroids (including oral budesonide, corticosteroid suppositories, foams, or enemas), immunomodulators, anti-tumor necrosis factor, biologics, anti-diarrheal, anti-depressant, anti-anxiety, narcotics, lactulose, probiotic, lubiprostone, and NSAIDs.

### Survey protocol

A modified survey questionnaire—used in a previous study—was mailed, relevant to gynecological and gastroenterological domains [9]. The survey included a list of symptoms that commonly occur around the menses, including upper and lower GI symptoms, psychological symptoms, and gynecological symptoms. Participants were asked to consider their current menstrual cycle and cycles in the recent months in order to identify which symptoms typically occurred during two phases in the menstrual cycle: (i) the premenstrual phase, which was defined as 1–5 days prior to menses, and (ii) the menstrual phase, which was defined as the days of their menstrual flow. Participants indicated whether the following symptoms were present or absent during these two periods; abdominal pain, pelvic pain, back pain, headache, breast pain, nausea, vomiting, diarrhea, urgency, mucus in stool, blood in stool, bloating, fever, fatigue, joint pain, depression/sadness, anxiety, irritability, nervousness, restlessness, swelling of legs, swelling of breast, acne, increase sexual drive and stress. They also indicated stool frequency and consistency. We also included intensity scales from 0–10 for pain variables, where 0 was defined as no pain and 10 as the worst pain ever experienced. The patients were further divided into two groups based on symptom subscore of mPDAI: the study group (GI symptom exacerbation during the menses) and control group (stable or better GI symptoms during menses). The mPDAI scores included abdominal pain, urgency, blood in stool, fever and diarrhea. Scoring was documented as follows: increase in pain intensity or the presence of symptom during pre-menses or menses was given a score of 1 and for diarrhea, the score was 2. Finally the mPDAI scores were compared between pre-menses and menses for every patient.

### Measurement of outcomes

The primary outcome of interest was the association of GI symptoms before and during menses in patients with IPAA. The secondary outcome was identifying factors associated with exacerbation of GI symptoms during menses in those patients.

### Statistical analysis

Descriptive statistics were computed for all variables. These included means and standard deviations (SD), or medians and interquartile ranges (IQR) for continuous variables and frequencies for categorical variables. Comparisons of the all symptoms, pre-menses and during menses, were made using the Wilcoxon signed-rank test for continuous variables and McNemar test for categorical variables. Comparison of patients' demographic and clinical features, with and without increased symptoms during menses, were made by using the two-tailed *t*-test (or the Fisher's exact test, as appropriate). A multivariate conditional logistic regression analysis using the forward stepwise method with an entry criterion of *P < *0.05 and a removal criterion of *P > *0.10 was applied, with the variable “pouch related disorders” being forced into the final model for the assessment of the factors associated with increased GI symptoms during menses. All statistical analyses were performed using SPSS software, version 16 (SPSS, Chicago, IL). A *P*-value less than 0.05 was considered statistically significant.

## RESULTS

**Figure 1.**
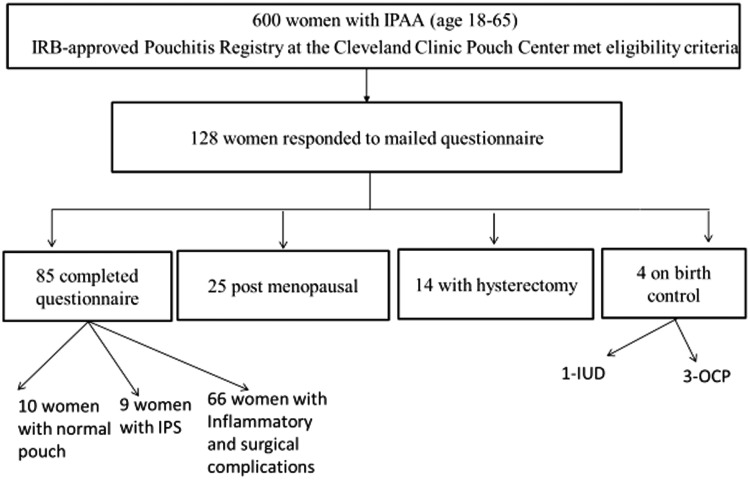
Flow diagram of enrolment of respondents with ileal pouch-anal anastomoses (IPAA).

Of the 600 women with IPAA invited to participate, 128 completed and returned the questionnaire, with a response rate being 21.3%. Forty three (33.6%) patients were subsequently excluded: 25 were post-menopausal, 14 had had a hysterectomy, and 4 used medical contraception. The final sample of 85 (66.4%) patients with ileal pouches were included; 62 with inflammatory conditions of the pouch (acute and chronic pouchitis, cuffitis and Crohn’s disease [CD] of the pouch), 10 with normal pouches, 9 with irritable pouch syndrome, 4 with pouch procedure-associated complications (Figure 1). The mean age was 38.0 ± 9.5 years. GI symptoms (Table 1), such as abdominal pain (median = 3.0; interquartile range [IQR]: 1.0, 6.0 for the study group *vs* median = 5.0; IQR 3.0, 8.0 for the control group; *P = *0.001), diarrhea (49.4% *vs* 61.2%; *P = *0.021), urgency (41.2% *vs* 52.9%; *P = *0.031), were more commonly reported during menses than pre-menses by the patients. Systemic symptoms (Table 1) like pelvic pain (median = 3.0; IQR 0.3, 6.0 *vs* median = 4.0; IQR 1.5, 7.0; *P = *0.007), back pain (median = 2.0; IQR 0.0, 5.0 *vs* median = 3.0; IQR 0.0, 7.0; *P = *0.02) were also reported worse during menses. However, other systemic symptoms such as breast pain (median = 2.0; IQR 0.0, 6.0 *vs* median = 1.0; IQR 0.0, 4.0; *P = *0.008), swelling of breast (50.6% *vs* 37.6%; *P = *0.043), increase sexual drive (58.8% *vs* 36.5%; *P = *0.001) were worse during pre-menses compared to menses. Psychological symptoms such as nervousness (27.1% *vs* 16.5%; *P = *0.022) and stress (65.9% *vs* 55.3%; *P = *0.035) were worsened during pre-menses.

**Table 1.** Association of gastro-intestinal symptoms and menstrual cycle (*n* = 85)

Characteristics	Pre-menses	During menses	*P-*value
mPDAS, median (IQR)	2.0 (0.0–3.0)	3.0 (1.0–4.0)	<**0.001**
**Gastro-intestinal symptoms**			
Abdominal pain, median (IQR)	3.0 (1.0–6.0)	5.0 (3.0–8.0)	**0.001**
Nausea, *n *(%)	14 (16.5%)	19 (22.4%)	0.13
Vomiting, *n *(%)	1 (1.2%)	1 (1.2%)	1.0
Diarrhea, *n *(%)	42 (49.4%)	52 (61.2%)	**0.021**
Urgency, *n *(%)	35 (41.2%)	45 (52.9%)	**0.031**
Mucus in stool, *n *(%)	19 (22.4%)	20 (23.5%)	1.0
Blood in stool, *n *(%)	11 (12.9%)	12 (14.1%)	1.0
Bloating, *n *(%)	58 (68.2%)	58 (68.2%)	1.0
Fever, *n *(%)	6 (7.1%)	2 (2.4%)	0.13
**Systemic symptoms**			
Pelvic pain, median (IQR)	3.0 (0.3–6.0)	4.0 (1.5–7.0)	**0.007**
Back pain, median (IQR)	2.0 (0.0–5.5)	3.0 (0.0–7.0)	**0.020**
Breast pain, median (IQR)	2.0 (0.0–6.0)	1.0 (0.0–4.0)	**0.008**
Headache, median (IQR)	1.0 (0.0–6.0)	2.5 (0.0–0.5)	0.4
Fatigue, *n *(%)	51 (60.0%)	57 (67.1%)	0.24
Joint pain, *n *(%)	35 (41.2%)	37 (43.5%)	0.63
Swelling of legs, *n *(%)	11 (12.9%)	9 (10.6%)	0.77
Acne, *n *(%)	48 (56.5%)	43 (50.6%)	0.27
Sexual drive, *n *(%)	50 (58.8%)	31 (36.5%)	**0.001**
Swelling of breast, *n *(%)	43 (50.6%)	32 (37.6%)	**0.043**
Depression and sadness, *n *(%)	32 (38.1%)	31 (36.9%)	1.0
Anxiety, *n *(%)	28 (32.9%)	24 (28.2%)	0.42
Irritability, *n *(%)	58 (68.2%)	50 (58.8%)	0.19
Nervousness, *n *(%)	23 (27.1%)	14 (16.5%)	**0.022**
Restlessness, *n *(%)	27 (31.8%)	22 (25.9%)	0.18
Stress, *n *(%)	56 (65.9%)	47 (55.3%)	**0.035**

mPDAS = modified pouch disease activity score; IQR = interquartile range

### Univariate and multivariate comparison of clinical and demographic features

Table 2 presents comparison of demographics, IBD, pouch-related variables, extra-intestinal manifestations, women-related variables such as length of menstrual cycle, days of menstrual flow, whether menses were painful, inter-menstrual bleeding, number of pregnancies, number of vaginal deliveries, number of caesarean sections, number of children, use of NSAIDs, use of OCP’s, education level and menopausal state. The patients were divided into two groups based on mPDAI score: the study group (*n = *46) and control group (*n = *39) to assess factors for worsening GI symptom score during menses. The mean age was 38.4 ± 10.4 years for study group and 37.5 ± 8.5 years for the control group. There was no significant difference in BMI between the groups (23.5 ± 5.2 kg/m^2^ *vs* 23.5 ± 5.6 kg/m^2^). There were more active smokers in the study group (6.7% *vs* 2.6%). The mean length of the menstrual cycle (27.7 ± 1.6 days *vs* 28.6 ± 3.3 days) and the mean menstrual period duration (5.6 ± 1.7 days *vs* 5.3 ± 1.2 days) did not differ between the groups. The use of antibiotics was reported in 23.9 % of the study group and 15.4% in the control group. The use of NSAIDs did not differ between the groups (13.0% *vs* 15.4%).

**Table 2.** Univariate analysis of factors associated with worsening of gastro-intestinal symptoms during menses

Characteristics		All patients (*n* = 85)	Patients without increased symptoms during menses (*n* = 39)	Patients with increased symptoms during menses (*n* = 46)	*P-*value
Age, years		38.0 ± 9.5	37.5 ± 8.5	38.4 ± 10.4	0.68
Body mass index, kg/m^2^		23.5 ± 5.4	23.5 ± 5.6	23.5 ± 5.2	0.95
Caucasian, *n *(%)		84 (98.8%)	38 (97.4%)	46 (100.0%)	0.46
Smoking, *n *(%)					0.21
Active		4 (4.8%)	1 (2.6%)	3 (6.7%)	
Ex-smokers		9 (10.6%)	6 (15.4%)	3 (6.5%)	
Never		72 (85.7%)	32 (82.1%)	40 (88.9%)	
Use of alcohol, *n *(%)		8 (9.4%)	4 (10.3%)	4 (8.7%)	1.00
**Pouch variables**					
Duration of IBD, years, median (IQR)		5.0 (2.0–7.0)	5.0 (2.0–8.0)	4.0 (1.8–6.3)	0.54
Duration of ileal pouch, years, median (IQR)		10.0 (5.0–16.0)	10.0 (5.0–15.0)	10.5 (6.8–17.0)	0.25
Baseline stool frequency, median (IQR)		7.0 (5.0–8.5)	6.0 (5.0–8.0)	7.0 (5.0–9.0)	0.15
Family history of IBD, *n *(%)		23 (27.1%)	8 (20.5%)	15 (32.6%)	0.21
Family history of colorectal cancer, *n *(%)		18 (21.2%)	8 (20.5%)	10 (21.7%)	0.89
Indication for colectomy, *n *(%)					1.0
Refractory		82 (96.5%)	38 (97.4%)	44 (95.7%)	
Neoplasia		3 (3.5%)	1 (2.6%)	2 (4.3)	
Extensive colitis, *n *(%)		78 (91.8%)	36 (92.3%)	42 (91.3%)	0.62
Fulminant colitis, *n *(%)		7 (8.2%)	4 (10.3%)	3 (6.5%)	0.7
Pouch configuration, *n *(%)					0.77
J-pouch		77 (90.6%)	37 (94.9%)	40 (87.0%)	
S-pouch		4 (4.7%)	1 (2.6%)	3 (6.5%)	
K-pouch		3 (3.5%)	1 (2.6%)	2 (4.3%)	
W-pouch		1 (1.2%)	0 (0.0%)	1 (2.2%)	
Stage of pouch, *n *(%)					0.56
1 stage		2 (2.4%)	0 (0.0%)	2 (4.8%)	
2 stage		58 (68.2%)	29 (74.4%)	29 (63.0%)	
3 stage		15 (17.6%)	7 (17.9%)	8 (17.4%)	
4 or redo		10 (11.8%)	3 (7.7%)	7 (15.2%)	
Any concurrent medications, *n *(%)		41 (48.2%)	19 (48.7%)	22 (47.8%)	0.94
Concurrent antibiotics, *n *(%)		17 (20.0)	6 (15.4%)	11 (23.9%)	0.33
Extra-intestinal manifestations, *n *(%)		12 (14.1%)	5 (12.8%)	7 (15.2%)	0.75
Pouch-related disorders, *n *(%)					0.93
Normal pouch		10 (11.8%)	5 (12.8%)	5 (10.9%)	
Irritable pouch syndrome		9 (10.6%)	5 (12.8%)	4 (8.7%)	
Inflammatory changes		62 (72.9%)	27 (69.2%)	35 (76.1%)	
Surgical complications		4 (4.7%)	2 (5.1%)	2 (4.3%)	
Non-steroidal anti-inflammatory drug, *n *(%)		12 (14.1%)	6 (15.4%)	6 (13.0%)	0.78
**Menstrual variables**					
Age at menarche, years	12.9 ± 1.4		12.7 ± 1.2	13.0 ± 1.6	0.28
Menstrual cycle duration, days	28.0 ± 2.9		28.6 ± 3.3	27.7 ± 1.6	0.2
Menstrual flow, days	5.5 ± 1.5		5.3 ± 1.2	5.6 ± 1.7	0.25
Number of pregnancy, median (IQR)	1.0 (0–2.0)		1.0 (0–2.0)	2.0 (0–2.0)	0.58
Number of children, median (IQR)	1.0 (0–2.0)		0.0 (0–2.0)	1.0 (0–2.0)	0.64
Menopause, *n *(%)	8 (9.4%)		2 (5.1%)	6 (13.0%)	0.28
Painful menses, *n *(%)	22/41 (53.7%)		6/19 (31.6%)	16/22 (72.7%)	**0.008**
Inter-menstrual bleeding, *n *(%)	7/36 (19.4%)		3/15 (20.0%)	4/21 (19.0%)	1.0

IBD = inflammatory bowel disease; IQR = interquartile range.

*Data on all patients were not available due to partial completion of questionnaires.

In the multivariate logistic regression model, only history of painful menses was associated with increased GI symptoms in patients with ileal pouch (OR = 5.67; 95% confidence interval [CI]: 1.41–22.88; *P = *0.015). Pouch disorder subtypes, such as irritable pouch syndrome (OR = 0.17, 95% CI: 0.005–5.86; *P = *0.33) and inflammatory conditions of pouch (OR = 0.74, 95% CI: 0.05–11.12; *P = *0.83) were not associated with increased GI symptoms during menses when compared to pre-menses.

## DISCUSSION

This study investigated the prevalence of GI symptoms in the menstrual cycle in patients with ileal pouches, and the factors associated with increased GI symptoms during menses, building on the clinical research that has started to examine these relationships in IBD. We found that GI symptoms were more frequent during menses, compared with pre-menses, for these women with IPAA. A history of primary dysmenorrhea was the only factor was found to be associated with increased GI symptoms during menstruation GI symptoms in menses. Interestingly, none of the pouch-specific or medication variables were associated with worsening of GI symptoms during menses. As for NSAIDs, being one of the treatment regimens for dysmenorrhea, our study was not powerful enough to investigate its role in GI symptomatology during menses.

Many healthy women experience variation in GI symptoms during the menstrual cycle [10]. It is difficult to distinguish prior to normal physiological variation from pathological exacerbation [15]. A survey conducted on 67 healthy volunteers found that 43 (64%) of them complained of change in bowel habit, either constipation or diarrhea, during active menstruation [15]. The association was also shown in IBS, where patients were more likely to experience increased flatulence, abdominal pain, change in bowel habits—either diarrhea or constipation—during menstruation than healthy controls [11]. In addition, a recent meta-analysis showed that 40–60% of women with IBS reported worsen GI symptoms during menses, predominantly diarrhea [12].

There are few studies that have investigated the association between menstrual cycle and IBD. A survey of 88 women with IBD, 49 with IBS and 90 healthy controls, found that women with CD were more likely to report worsening of GI symptoms during menses [13]. Also, IBD patients were more likely than healthy controls to report a cyclical pattern to their bowel habit, with recurrence of diarrhea during menses [19]. Another study, which compared 151 patients with CD, 87 patients with UC and 156 healthy controls, showed worsening of GI symptoms in IBD patients during menses [9]. The above studies were limited due to their retrospective nature. To address this issue, two studies used daily dairies to prospectively study the pattern of GI symptoms during the menstrual cycle. One study compared 47 IBD patients (27 UC and 13 CD) with 44 healthy controls and found increased GI symptom scores in UC patients and healthy controls during menstruation, when compared with pre-menses [10]. The second study prospectively evaluated 97 subjects, 59 with IBD and 38 healthy controls. Patients in both IBD and control groups reported worse GI symptoms during menses than pre- or post-menses [14].

Currently there are scant data on the menstrual cycle relating to GI symptoms in pouch patients. In this study, we found worsening of GI symptoms which included diarrhea, abdominal pain, and urgency, as evidenced by the higher mean mPDAI scores during menses. The findings of our current study supports the above notion on the association of menstruation and GI symptoms in IBD. The onset of menstruation is associated with an increase in uterine PGs, particularly PGF2α and prostacyclin, which are well known to have a powerful stimulatory effect on both the motor and secretory activity of the gut [15]. These PGs therefore exert GI actions if they escape degradation and gain access to the systemic circulation [22]. Further, dysmenorrhea and menstrual migraine are common problems experienced by women of reproductive age. Dysmenorrhea is linked to excess or imbalance of PGs and arachidonic acid metabolites in uterine, myometrial and/or vascular smooth muscle, causing increased contractions [16]. As the GI tract is also composed of smooth muscle, excessive PGs in the blood may alter the function of this tissue. Evidence supporting the association between menstruation, PG release and diarrhea is derived from clinical studies on the therapeutic effect of PG inhibitors, including NSAIDS, in patients with primary dysmenorrhea [17].

Additionally, we also found that, patients with IPAA reported worsening of pelvic pain during menses. Rectal sensitivity (induced by distention of a rectal balloon controlled by a barostat) was shown to be increased at menses compared with all other cycle phases, consistent with the hypothesis that reduced or decreased ovarian hormones may contribute to increased pain sensitivity during menses [18].

In our study, symptoms such as breast pain, breast swelling, nervousness, stress and increased sexual drive were more commonly reported prior to than during menstruation. This is supported by a study that surveyed 88 women with IBD, 49 with IBS, and 90 healthy controls and found that 93% of their patients reported at least one premenstrual symptom [19]. A central role of ovarian sex hormones in the etiology of premenstrual symptoms is strongly supported by a series of experiments in which ‘medical oophorectomy’—using a gonadotropin-releasing hormone agonist—led to dramatic resolution of the symptoms [19]. Also there is evidence that cyclic fluctuations in circulating estrogen and progesterone cause marked changes in the opioid, gamma-aminobutyric acid (GABA), and serotonin systems [25–27], and that, due to the presence of serotonin receptors in the GI tract, fluctuations in gonadal hormones influence pain sensitivity.

Our study is clinically significant for the following reasons: first, our results substantiate the findings of previous studies, in which a majority of patients with IBD reported worsening of symptoms during menses, as compared with pre-menses. However, whether or not the symptoms resolve during the post-menstrual phase could not be determined from our study design. Second, painful menses was found to be a factor for worsening GI symptoms. As previously mentioned, dysmenorrhea has been linked with excess PG during menses, and leakage of PG into the systemic circulation can cause worsening of GI symptoms. The result is further supported by a recent study which found that dysmenorrhea was highly prevalent in women with CD, occurring in about 40% of cases [20].

Our study might be underpowered to find any association of NSAID use, painful menses and GI symptomatology. In our previous study, we found that NSAID use was associated with the development of pouchitis [21]; however, a short course of PG-targeted NSAIDs may be beneficial in relieving menses-associated GI symptoms in selected patients, based on the results of our current study.

There are several limitations to this study. First, it might have incurred referral bias, as the majority of patients in our Pouchitis Clinic had mechanical, functional, inflammatory pouch disorders. However, the sub-specialty clinic has made it possible to assess a population with diverse disease conditions of the ileal pouch. Second, the response rate to our study was low (21.3%), despite reminder phone calls, which has led to high non-response bias and also selection bias. We now believe that mixed-mode approach, combining mailed and e-mailed surveys, reminder phone calls and rewards for answering the survey, would have yielded better response rates. Third, responses may also have been subject to recall bias, as women were not uniformly surveyed at the same point in the menstrual cycle; hence it is possible that individual recall may have been different depending on the time in their cycle when they each responded. nevertheless, they were asked to consider recent months when responding, not just the current cycle. Fourth, due to the retrospective nature of the study, patients with severe symptoms may have been more likely to respond to the questionnaire than those with mild-to-moderate disease. Furthermore, there is an issue of inter-individual variability when assessing the consistency of the symptoms reported. Future studies involving daily dairies would negate the recall bias. Fifth, we included a heterogeneous pouch patient population with diagnosis ranging from normal to diseased pouch, which may have confounded our findings in worsening GI symptoms during menses. Sixth, we did not assess menstrual symptoms specifically in relation to pouchitis disease activity. Seventh, we included few post-menopausal women in the analysis due to the low rate of response to our study. The perimenopausal symptoms in some of these women could have further confounded our results. However, despite these limitations, Cleveland Clinic Pouchitis Registry is one of the largest pouch patient databases and, despite the low response rate, our study is the first to investigate the variation of GI symptoms during the menstrual cycle in this patient population. Finally, the results of our study would be a stepping-stone for future prospective studies to elucidate the mechanisms of this association and to understand the impact of pelvic surgery on menstrual symptoms.

In conclusion, IPAA patients appeared to have worsening of GI symptoms during menses. Painful menstruation was found to be associated with worsening of GI symptoms. Knowledge of the variations in GI symptoms in patients with ileal pouch during the menstral cycle may be helpful in differentiating normal physiological variations from disease exacerbation and aid in future therapeutic intervention.
